# Mechanical Structural Design of a MEMS-Based Piezoresistive Accelerometer for Head Injuries Monitoring: A Computational Analysis by Increments of the Sensor Mass Moment of Inertia †

**DOI:** 10.3390/s18010289

**Published:** 2018-01-19

**Authors:** Marco Messina, James Njuguna, Chrysovalantis Palas

**Affiliations:** 1 Maritime and Mechanical Engineering Department, Liverpool John Moores University, James Parsons Building Byrom Street, Liverpool L3 3AF, UK; palasv@hotmail.com; 2 Centre for Advanced Materials Engineering, School of Engineering, Robert Gordon University, Aberdeen AB10 7GJ, UK; j.njuguna@rgu.ac.uk

**Keywords:** piezoresistive accelerometer, sensor design, biomechanical device, head injuries monitoring, TBI

## Abstract

This work focuses on the proof-mass mechanical structural design improvement of a tri-axial piezoresistive accelerometer specifically designed for head injuries monitoring where medium-G impacts are common; for example, in sports such as racing cars or American Football. The device requires the highest sensitivity achievable with a single proof-mass approach, and a very low error (<1%) as the accuracy for these types of applications is paramount. The optimization method differs from previous work as it is based on the progressive increment of the sensor proof-mass mass moment of inertia (MMI) in all three axes. Three different designs are presented in this study, where at each step of design evolution, the MMI of the sensor proof-mass gradually increases in all axes. The work numerically demonstrates that an increment of MMI determines an increment of device sensitivity with a simultaneous reduction of cross-axis sensitivity in the particular axis under study. This is due to the linkage between the external applied stress and the distribution of mass (of the proof-mass), and therefore of its mass moment of inertia. Progressively concentrating the mass on the axes where the piezoresistors are located (i.e., *x*- and *y*-axis) by increasing the MMI in the *x*- and *y*-axis, will undoubtedly increase the longitudinal stresses applied in that areas for a given external acceleration, therefore increasing the piezoresistors fractional resistance change and eventually positively affecting the sensor sensitivity. The final device shows a sensitivity increase of about 80% in the *z*-axis and a reduction of cross-axis sensitivity of 18% respect to state-of-art sensors available in the literature from a previous work of the authors. Sensor design, modelling, and optimization are presented, concluding the work with results, discussion, and conclusion.

## 1. Introduction

One way of converting acceleration in an electrical signal is to deploy the piezoresistive effect. When the proof-mass of an accelerometer is affected by inertial forces due to an external acceleration the strain/stress on the piezoresistors determines displacement that consequently produces a change in its resistance value proportionally to the acceleration applied to the device. Therefore, the voltage output will change accordingly and will represent, by less than a constant of proportionality, a measure of the acceleration.

Currently, when designing an acceleration sensor, the main struggle for designers is to find the right trade-off between sensor sensitivity and size, because the sensitivity significantly drops with size. Today’s technology limits the size of an accelerometer below 1 square millimeter for the extreme loss in sensitivity [1,2]. Clearly when miniaturization is an objective of the design, sensitivity becomes the main issue, because a reduced sensitivity will severely affect the device accuracy due to a low signal-to-noise ratio. In order to address this sensitivity problem, a simple but ineffective solution is to introduce an amplifier at the output level. This results in adding additional signal noise that certainly deteriorates the accuracy of the measurement.

This work aims at the design optimization of the mechanical structure of a piezoresistive accelerometer specifically designed for high performance traumatic brain injury (TBI) measurement. The challenges faced in this particular design are miniaturization combined with high sensitivity and lowest possible cross-axis sensitivity (error from other axes of measurement). Notice that all the other sensor specifications—such as resolution, noise, temperature drift, etc.—which are related to the electrical piezoresistors design, are not covered in this work as only the device mechanical structure is studied here. The piezoresistors deployed for the sensor performance calculations are conventional micrometer devices.

When a head injury occurs, especially in helmeted sports such as race car accident, TBIs are very common. In the past decade, considerable research efforts have been made in order to prevent and monitor the severity of head injuries, especially in motorsports. Many accidents and also deaths of race drivers occurred without any type of monitoring in the past, therefore, links between accelerations of specific body parts and injury could not be thoroughly made.

In the late 90s, an initial solution was introduced in the form of helmet-mounted sensors measuring crash severity with the help of accelerometers. However, the solution was found to be not very appropriate because these instrumented helmets may not accurately measure the actual acceleration experienced by the head due to helmet-to-head fit and helmet liner properties [3,4,5,6,7,8,9]. Therefore, it was very difficult to estimate the acceleration forces passed to the head, because the helmets are designed to minimize the amount of acceleration experienced by the head, and in this way the acceleration measured by this technology may not reflect the head acceleration [10,11,12].

Further studies in this area suggest that, for an accurate detection of head acceleration, that the coupling between head and sensor is crucial, therefore the instrumented helmet solution was soon replaced in the new century by a mouthpiece accelerometer in football [13] and by an accelerometer attached to an earpiece and not to the helmet in motorsports [14,15]. These novel solutions allowed a direct and therefore more accurate assessment of head acceleration.

In 2003, a version of this type of earpiece with an integrated acceleration sensor, called the Delphi Earpiece Sensor System (DESS) [16], was introduced for the first time in the Indy Racing League and Championship Auto Race Teams (CART). In 2006, a group of researchers at the Wayne State University—led by Begeman [17]—reported that these earplugs mounted in post mortem human specimens (PMHS) showed in the output signal a progressive phase lag from 50 to 100 Hz vibration when compared to skull measurement (rigidly mounted head accelerometers).

Furthermore, in 2009, Salzar et al. [18] explored a solution in order to try to avoid the issue found by Begeman earlier by developing a smaller tri-axial device meant to be placed inside the ear canal portion of the earpiece. The sensor showed improved coupling to the head over the DESS that was perceived to be too bulky [19]. Salzar adds that to further enhance the accuracy of the measurements it is advisable to improve the positioning technique and the mounting material, basically a stiffer material is recommended [18]. However, the sensor accuracy and miniaturization obtained by Salzar is not yet acceptable for this type of in situ ear measurement. It is expected that an acceptable sensor error should be below 1% combined with a miniaturization below 2 × 2 mm^2^.

In 2013, an attempt was made to improve earplug sensor sensitivity and miniaturization by integrating silicon nanowires as nanoscale piezoresistors. However, manufacturing limitations prevented successful fabrication of a proof of concept [20,21,22]. Finally, in 2014, a patent was published for a novel optimization method based on variation of the sensor mass moment of inertia [20,23].

This work attempts a further improvement of the authors patented work on earplug sensor technology, (patent number EP2741088A9 published at the EPO [23]), by investigating a way of enhancing sensor performances and miniaturization by specific increments of the sensor mass moment of inertia (MMI), with the objective of achieving the most accurate response in case of medium-G impact crashes (<500 G) not yet being achieved in the state-of-art sensor design. The data gathered with a more accurate sensor would benefit all stakeholders involved in the motorsport community and industry, for example by helping to design better car safety restraint systems, like shoulder harnesses, helmets, seat belts, and head and neck restraints commonly used in all forms of racing.

## 2. Theory and Method

### 2.1. Theory of Operations

The piezoresistive effect is expressed by a matrix where each of the six fractional resistivity changes relates to each of the six stress components [24]. The Kanda [25] general equation of the fractional resistivity change with small stress is expressed as in Equation (1) and mathematically this produced a matrix of 36 coefficients [24]

(1) $$\frac{\Delta \rho_{\omega }}{\rho }=\overset{6}{\underset{\lambda =1}{\sum }}\pi_{\omega \lambda }\times \sigma_{\lambda }$$

where ρ is the resistivity, $\pi_{\omega \lambda }$ is the component of the piezoresistance tensor, $\sigma_{\lambda }$ is the component of the stress tensor, *ω* is both a fixed voltage and current orientation, and *λ* is the stress orientation.

The coefficients are called piezoresistance coefficients, $\pi_{\omega \lambda }$, (*ω*, *λ* = 1 to 6), and are expressed in Pa^−1^. In crystals with cubic symmetry, such as silicon and germanium, the tensor is given by [25]

$$\left[\pi_{\omega \lambda }\right]=\left[\begin{array}{cccccc} \pi_{11} & \pi_{12} & \pi_{12} & 0 & 0 & 0\\ \pi_{12} & \pi_{22} & \pi_{12} & 0 & 0 & 0\\ \pi_{12} & \pi_{12} & \pi_{33} & 0 & 0 & 0\\ 0 & 0 & 0 & \pi_{44} & 0 & 0\\ 0 & 0 & 0 & 0 & \pi_{44} & 0\\ 0 & 0 & 0 & 0 & 0 & \pi_{44} \end{array}\right]$$

Typically, each piezoresistor has two contacts that are made by masked-ion implantation method and located on the beam, which is a very thin surface layer [26]. Thus, for the purpose of calculation only two piezoresistive coefficients are relevant, defined by Mason in [27] as longitudinal (π′_11_) and transverse (π′_12_) piezoresistive coefficient. In the particular case that the stress is parallel with the direction of electric field and current density we use π′_11_, that is called the longitudinal piezoresistance coefficient, denoted by π_l_. Likewise, in case the applied stress is perpendicular to the electric field and current density we use π′_12_, therefore called transverse piezoresistance coefficient, π_t_. The shearing stress is neglected since it is much smaller than the others.

Mason [27] derived directional coefficients from full formulations relating the electric field, current density, and stress components. He also presented more general formulations of these two coefficients through three fundamental piezoresistance coefficients—π_11_, π_12_, π_44_—and directional cosines (l, m, n) for arbitrary crystal orientation, expressed by

(2) $$\pi_{11}^{\prime }=\pi_{11}-2\pi_{ο}\left(l_{1}^{2}m_{1}^{2}+l_{1}^{2}n_{1}^{2}+m_{1}^{2}n_{1}^{2}\right)$$

(3) $$\pi_{12}^{\prime }=\pi_{12}-\pi_{ο}\left(l_{1}^{2}l_{2}^{2}+m_{1}^{2}m_{2}^{2}+n_{1}^{2}n_{2}^{2}\right)$$

(4) $$(\pi_{ο}=\pi_{11}-\pi_{12}-\pi_{44})\;\mathrm{and}\;{\pi^{\prime }}_{12}={\pi^{\prime }}_{21}$$

Thanks to the theory and equations above, the resistance change becomes a function of the beam stress. This is true because in the real situation the piezoresistors are located on the thin beam surface, therefore at the surface plane the material is stressed mainly in two directions. Given the assumption that the mechanical stresses are constant over the piezoresistors, the fractional resistance change is given by

(5) $$\frac{\Delta R}{R}=\sigma_{l}\pi_{l}+\sigma_{t}\pi_{t}$$

where σ_l_ and σ_t_ are longitudinal and transversal stress.

It is worth noting that the Equation (5) is only useable for uniform stress fields or if the piezoresistor dimensions are small compared to the beam size [24,28].

Single crystal germanium and silicon are the first materials extensively used as piezoresistors due to their diamond lattice crystal structure. In 1954, Smith [29] tested these semiconductor crystals and for the first time a large piezoresistive effect was reported observing that this phenomenon is theoretically explained by the study undertaken by Bardeen and Shockley [30], and later Herring [31,32]. The work of Smith allowed the measurement of piezoresistive coefficients for (100)-silicon wafer along the <100> and <110> crystal orientations. Shear piezoresistive coefficients were indirectly calculated, whereas longitudinal and transverse coefficients were measured directly. In particular, Smith with these measurements fully determined the piezoresistive tensor at a resistivity of 7.8 Ω-cm at low p-Si concentration considering also the crystal symmetry. Finally, he found the p-type longitudinal piezoresistive coefficient in the [110]-direction at light boron concentrations (≈1.7 × 10^15^ cm^−3^) to be fairly constant at 72 × 10^−11^ Pa^−1^. Kanda later presented his results graphically [25].

From their findings, it can be asserted that p-type piezoresistors have to be oriented along the <110> directions to measure stress in (100)-wafers and thus the piezoresistors should be either lined up or perpendicular to the wafer primary flat [33]. These piezoresistor orientations are used in this work, moreover, for calculation of sensor performance the piezoresistive coefficient used is the one found by Smith [29] for p-type silicon at room temperature (Table 1).

In reality, the piezoresistive coefficients of single-crystal silicon varies and is dependent on the type of dopant [34], the doping concentration [34,35], and the temperature of the wafer substrate [25,34]. As a consequence, designers need to take into account temperature and doping concentrations at the design stage because many components of the π matrix (π_11_, π_12_, and π_44_) are affected in different ways. In particular, when the temperature and doping concentration increases the value of the piezoresistive coefficient decreases, and this behavior has been observed for both p- and n-type silicon. Under certain typical doping concentration and dopant types the values of π_11_, π_12_, and π_44_ for single-crystalline silicon have been experimentally determined. Table 1 below lists typical values for selected doping concentrations [29].

However, generally speaking, there are circumstances where all 36 coefficients in the matrix [π] may be nonzero [36] when referring to a Cartesian system of arbitrary orientation relative to the crystallographic axes. For silicon, if the *x*-, *y*-, and *z*-axes are not in line with <100> directions the matrix components change.

Instead, in specific conditions, where the piezoresistors points in <100>, <110>, or <111> directions [25,37] the effective longitudinal and transverse piezoresistive coefficients can be summarized as in Table 2.

Replacing the results in Table 1 into the formulas of the piezoresistive coefficients in Table 2 an estimation of the fractional resistance change given in Equation (5) for p-type and n-type piezoresistors in the <110> and <100> direction is achievable. For example, for n-type piezoresistors in the <100> direction with a resistivity of 11.7 Ω-cm the fractional resistance change is

(6) $$\frac{\Delta R}{R}=\sigma_{l}\pi_{11}+\sigma_{t}\pi_{12}=-\sigma_{l}102.2+\sigma_{t}53.4\approx \sigma_{l}\pi_{11}-\sigma_{t}\frac{\pi_{11}}{2}=\pi_{11}\left(\sigma_{l}-\frac{\sigma_{t}}{2}\right)$$

As it can be seen from Equation (6), the fractional resistance change is only a function of the longitudinal piezoresistive coefficient and when the transversal stress is doubled, the longitudinal stress it is zero. Therefore, this results shows the suitability of the n-type piezoresistors in the <100> direction for measuring acceleration when the main stress component is the longitudinal stress as in uniaxial stress applications. Clearly, the p-type piezoresistors in the same direction with a resistivity of 7.8 Ω-cm are not suitable for measurements given the very low piezoresistive coefficients.

Comparing the n-type piezoresistors in the <110> direction with a longitudinal coefficient of −31.2 × 10^−11^ Pa^−1^ and a transversal coefficient of −17.6 × 10^−11^ Pa^−1^, it is concluded that this configuration is generally not preferred for measurements on the <100> direction.

Instead in the <110> direction p-type piezoresistors with a resistivity of 7.8 Ω-cm show a fractional resistance change as

(7) $$\frac{\Delta R}{R}=\sigma_{l}\frac{\pi_{44}}{2}-\sigma_{t}\frac{\pi_{44}}{2}=\frac{\pi_{44}}{2}\left(\sigma_{l}-\sigma_{t}\right)$$

where compared to the π_44,_ π_11_, and π_12_ are considered zero due to their very low value.

This is the preferred configuration used in this study mainly because p-type piezoresistors in the <110> direction is a convenient crystallographic orientation from a fabrication standpoint [38], moreover boron is the most common used dopant. In a (100)-oriented wafer, the p-type piezoresistors in the <110> direction are perpendicular to each other, therefore it is possible from a design point of view to fabricate the piezoresistors in the *x*- and *y*-axis pointing to the <110> direction and detect the in-plane acceleration by simple Wheatstone Bridge circuits.

### 2.2. Design, Modelling, and Optimization

**Figure 1:**
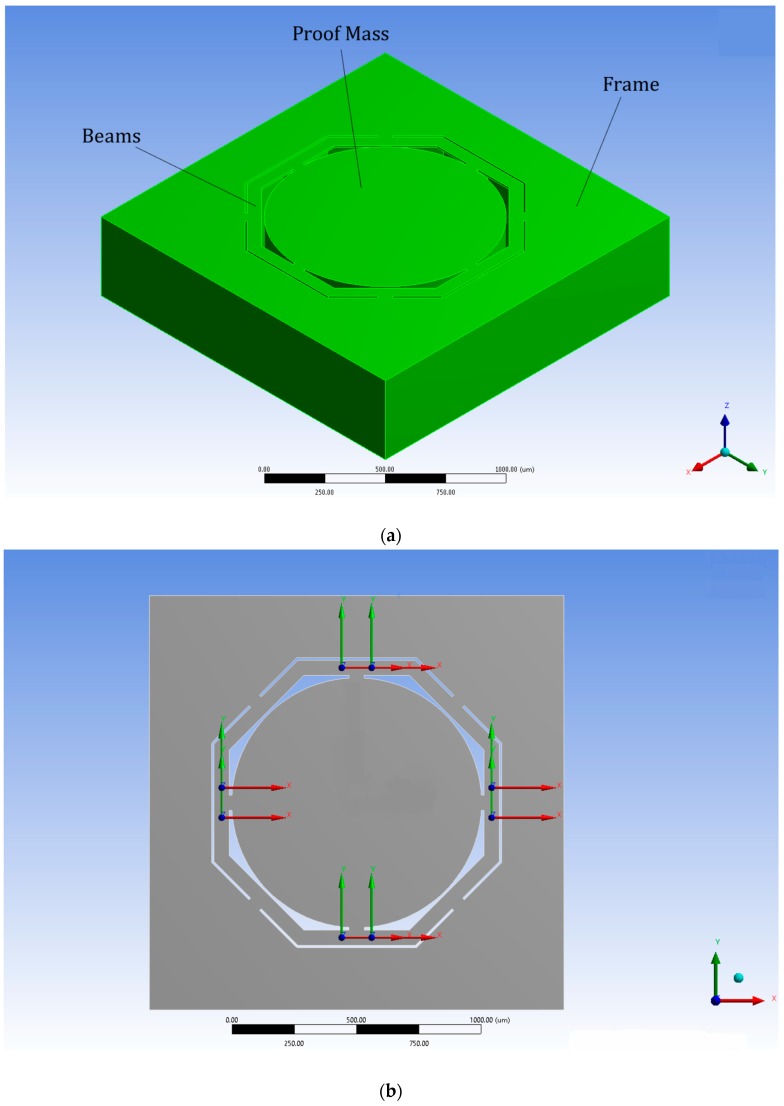
Mechanical structure of a triaxial accelerometer available in the literature by the first author Ph.D. thesis and patent [20,23]. (**a**) Isometric view with annotation of parts; (**b**) top view with location of piezoresistors.

The section describes the design, modelling, and optimization of a triaxial single square millimeter bio-mechanic piezoresistive accelerometer available from the literature as state-of-art device [20,23] and presents mass moment of inertia results. This chosen device as starting point of the optimization process is a triaxial accelerometer with one single mass available for all axes of measurement and it is characterized by a cylindrical proof-mass suspended by four octagonal beams fixed to an external frame (Figure 1a isometric view and Figure 1b top view) [20,23]. As accurate measuring of head accelerations is an important aspect in predicting head injury, it is important that the measuring sensor be well-coupled to the head [17]. Therefore, the main requirements of this application are miniaturization (≈1.5 × 1.5 mm^2^) and medium-G measurement range (<500 G) to allow the accelerometer incorporation into an earpiece. Bandwidth specification of the device is not relevant in this study as it can be adjusted by changing the device size accordingly. Typically, the frequency response of a miniature device like the one under study is of 1 kHz, a smaller device will determine a higher bandwidth of frequency. In reality for the particular application under study where high speed impacts are common, long duration transience is usually measured, therefore very low signal frequencies are expected in the order of 0–0.5 Hz. These low frequencies responses down to DC (i.e., they respond to steady-state accelerations) are specifically detected using a piezoresistor pick-off technology. At frequencies close to 0 Hz, piezoelectric accelerometers cannot, when high accuracy is required, measure the acceleration that an object is subject to [39].

**Figure 2:**
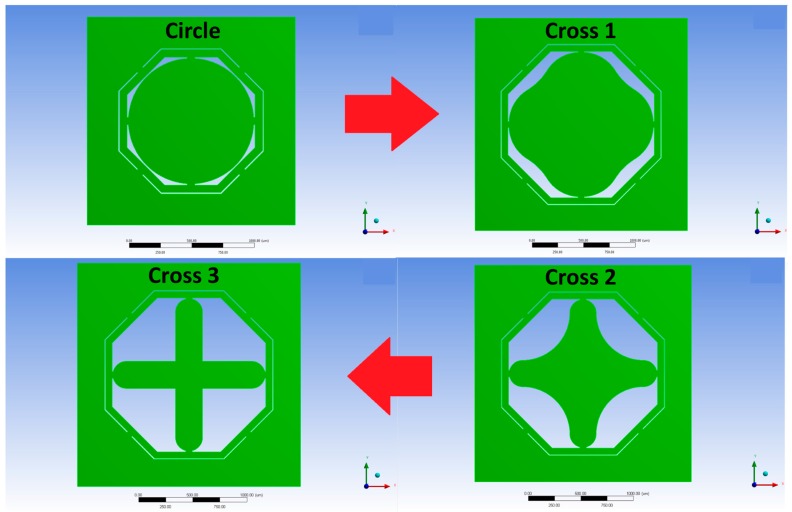
Mechanical structures top views. Optimization process increases the MMI at each step of evolution and therefore hypothetically there would be an increase in the sensitivity and a reduction in cross sensitivity.

In order to fulfil these requirements, high sensitivity is a paramount feature due to the small dimensions of the device under study. Moreover, minimizing the cross-axis sensitivity is also very important such that the acceleration measured on one axis is not mixed with errors coming from the other axes. As a rule of thumb, cross-axis sensitivity needs to be below 1% of the main signal coming out from the axis under stress in order to have an accurate measurement. The optimization methodology adopted in order to increase sensor sensitivity and minimized cross-axis sensitivity is based on the hypothesis that an increment of sensor MMI in all three axes will positively affect the sensor sensitivity and negatively influence the sensor cross-axis sensitivity, therefore improving overall sensor performance. This hypothesis is based on the linkage between the applied stress and the distribution of mass (of the proof-mass), and therefore of its mass moment of inertia. Stress distribution is function of mass distribution, and MMI is also a function of mass distribution, therefore stress distribution is function of MMI. An increment of MMI will determine a concentration of stress. Therefore, progressively concentrating the mass on the axes where the piezoresistors are located (i.e., *x*- and *y*-axis, see Figure 2) by increasing the MMI in the *x*- and *y*-axis, will undoubtedly increase the longitudinal stresses applied in that areas for a given external acceleration and therefore, increasing the piezoresistors fractional resistance change (see Equation (7)) and positively affecting the sensor sensitivity (see Equation (9)). On the other hand, the cross-axis sensitivity is reduced because of a decreasing concentration of mass in the proof-mass corner areas, responsible for producing unwanted combined biaxial accelerations for a given uniaxial acceleration applied.

The state-of-art sensor has been obtained from different optimization method based on MMI change as well [20,23]. In this study, the MMI will be increased progressively from a circular proof-mass design (Figure 1) to a cross design, that, at each of the three iteration of optimization, increases the angle of curvature of the proof-mass corners, until it becomes a complete cross as shown in Figure 2. The proof-mass design change from Circle to Cross 1 (top-right in Figure 2), and then to Cross 2 (bottom-right) and finally to Cross 3 (bottom-left).

Hypothetically, this optimization would particularly increase the sensor sensitivity and minimize the cross-axis sensitivity as the optimization reduces the distribution of mass on the biaxial area (*x*/*y*-axis) but increases on the single axial area (*x*- or *y*-axis), therefore increasing the MMI at each step of the design evolution. As explaining earlier, the increments of MMI will affect the proof-mass design by progressively increasing the concentration of the mass in the axes rather than in the corners. This evolution of the proof-mass design due to increments of the MMI in three steps of evolution (Figure 2) directly affects the stress distribution on the piezoresistors placed in the nearby locations on the suspended beams. This concentration of longitudinal stresses on the piezoresistors is demonstrated by higher stresses calculated by finite element analysis on those locations. Higher longitudinal mechanical stresses on the piezoresistors are directly beneficial to the sensor electrical sensitivity, as stated in Equation (9), because they will increase the change in resistance for a given external applied acceleration.

**Figure 3:**
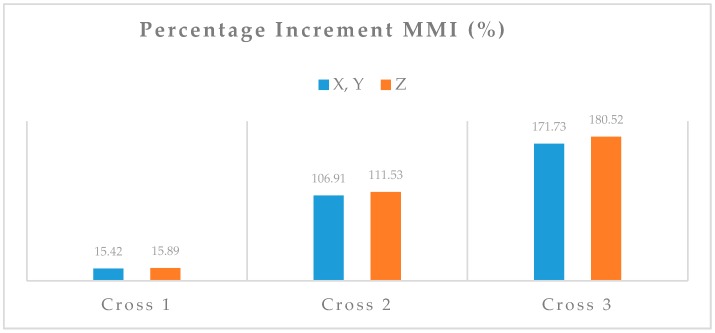
Percentage increment of MMI respect to the original circular proof-mass device. The design Cross 3 offers the highest percentage increment of MMI.

Figure 3 shows the percentage increment of the MMI of each new design in the *x*- or *y*-axis and in the *z*-axis compared to the state-of-art design (circle proof-mass) available in the literature. As can be seen, in order for the designs to be comparable the proof-mass volume of the pair of design under study needs to be the same value.

The values of mass moment of inertia of different proof-mass designs have been obtained from the CAD software ANSYS. The only way to define a precise MMI value for each design was to follow a trial and error design process, as the complexity of the designs could not precisely define a wanted MMI for each geometry. Therefore, we decided to gradually increase the curvature of the proof-mass in each corner (Figure 2) and to obtain the correspondent MMI. Obviously, the method is not very accurate, as can be seen the increments of MMI is not linear form one design to another, but it is effective for the aim of the research (to demonstrate correlatation between sensor sensitivity and MMI).

### 2.3. Measurement Circuit

Wheatstone Bridge [40] is formed by four resistors connected in a quadrangle. The excitation that could be voltage or current is connected across one diagonal, whereas in the other diagonal, there is a voltage detector. Basically, the detector measures the voltage output difference of two dividers connected to the excitation [26]. There are different configurations of the bridge circuit, but the best one that minimizes the nonlinearities and presents higher sensitivity is the full-bridge configuration which is also adopted in this study. In this configuration, the voltage output is simply the excitation voltage times the fractional resistance change, as in Equation (8).

(8) $$V_{out}=V_{in}\times \left(\frac{\Delta R}{R}\right)$$

**Figure 4:**
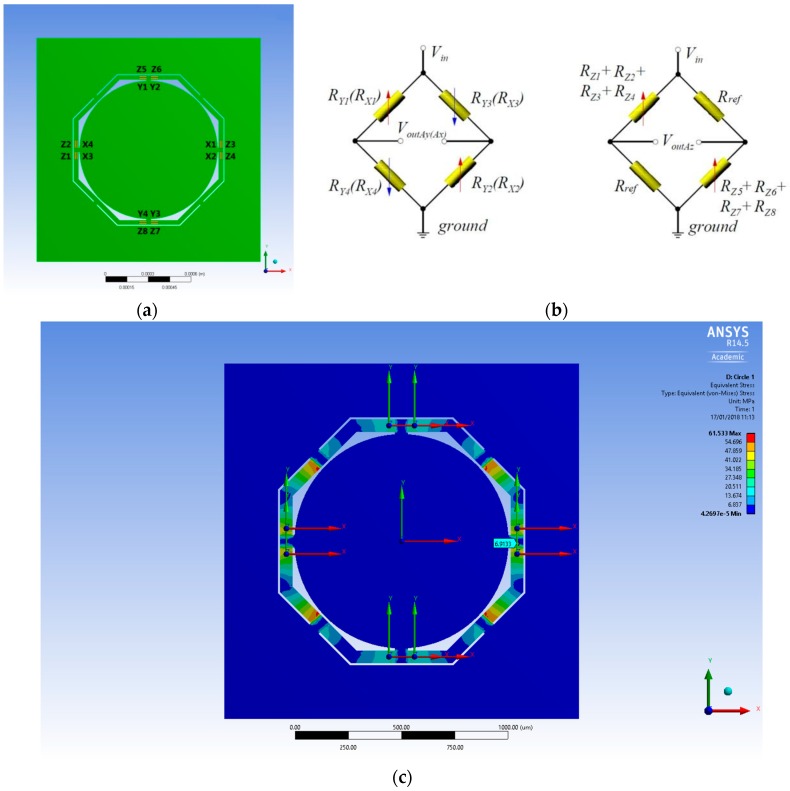
Measurement circuit design: (**a**) Piezoresistors location on the top surface of the device. A total of 16 piezoresistors are used, 4 for the *x*-axis, 4 for the *y*-axis, and 8 for the *z*-axis; (**b**) *Ax*-, *Ay*-, and *Az*-Wheatstone Bridge measurement circuit; (**c**) Stress distribution under *x*-axis acceleration. Piezoresistors are placed where the highest stress is located by the finite element stress distribution analysis in order to maximize sensor sensitivity (see axial coordinates). Notice that in the oblique directions on the beams, where there is a clear high stress distribution (in red), the piezoresistors cannot be placed as in these directions they do not show high piezoresistance effect.

In this work, four piezoresistors are used for the full-bridge for the *x*- or *y*-axis and eight piezoresistors are used for the *z*-axis, therefore a total of 16 piezoresistors are used and placed in strategic locations on the top surface of the device mechanical structure (Figure 4a). In particular, these piezoresistors are placed where the highest stress is located by the stress simulation analysis in order to maximize sensor sensitivity. These regions are identified by finite element stress distribution analysis, Figure 4c.

In Figure 4b, the three Wheatstone Bridges specifically designed to maximize sensor sensitivity are presented, one for each axis that measure the output voltage drop. The bridges have some advantages, in fact, by using similar resistors the balanced configuration allows for temperature drift cancellation. Moreover, thanks to the particular sensor design used, which is the highly symmetric geometry, self-cancellation of part of the cross-axis acceleration is possible. That is why the piezoresistors are placed symmetrically one another.

### 2.4. Electrical Sensitivity and Cross-Axis Sensitivity

In this work electrical sensitivity and cross-axis sensitivity have been selected as the mechanical structural parameters in order to measure the device performance. In the design of the micro-electro-mechanical device they give us information of accuracy and error in the measurements. The electrical sensitivity depends on the piezoresistors’ fractional resistance change, higher variation of resistance for a given applied external acceleration will positively affect the sensor sensitivity. The fractional resistance change depends on two parameters, the longitudinal piezoresistance coefficient (its value depends mainly of level and type of doping) and longitudinal and transversal stresses (their values depends on the mechanical design of the sensor structure) (Equation (11)). The same principles apply for the cross-axis sensitivity, its value is very much linked to the mechanical structural design. Structural symmetry minimizes sensor cross-axis sensitivity, and even more for a highly symmetric sensor geometry (symmetry on *x*- and *y*-axis, symmetry on the diagonal and symmetry on every 90° of rotation) such as our design. Therefore, sensor sensitivity and cross-axis sensitivity are used for main sensor mechanical structural parameters.

The accelerometer electrical sensitivity *S* is the ratio between the output voltage and the applied acceleration, as in Equation (9). When there are in-plane accelerations (*x*- or *y*-axis), the stress along the beams is perpendicular to the direction of the applied acceleration and represents the highest value respect to the stress distributed on the beam but parallel to the direction of the acceleration. Therefore, the piezoresistors to measure the acceleration on the *x* direction (*Ax*) are arranged on the *y*-oriented beams, and vice versa. For example, for the *Ax*-bridge in the case of *x*-axis acceleration, the electrical sensitivity is [41]

(9) $$S_{Ax}=S_{Ay}=\frac{Vout_{Ax}}{A_{x}}=\frac{1}{A_{x}}\times \frac{\Delta R_{x}}{R_{x}}\times V_{in}$$

where *V_out_* is the output voltage, *V_in_* is the bias voltage applied to the piezoresistor (5 V), and $\frac{\Delta Rx}{Rx}$ is the fractional resistance change of *Ax*-bridge that is equal to [24,28]

(10) $$\frac{\Delta R_{x}}{R_{x}}=\pi_{l}\times \sigma_{l}^{y}+\pi_{t}\times \left(\sigma_{t}^{x}+\sigma_{t}^{z}\right)$$

where π_l_ and π_t_ are longitudinal and transverse piezoresistive coefficients respectively and $\sigma_{l}^{y}$, $\sigma_{t}^{x},\sigma_{t}^{z},$ are respectively longitudinal stress in the *y*-axis in case of acceleration along the *x*-axis, and transverse stress in the *x*- and *z*-axis directions. Equation (10) is only valid for uniform stress fields or if the piezoresistor dimensions are small compared to the beam size [42].

Since the common approximation, where π_l_ = −π_t_ is valid in the <110> silicon crystallographic direction, the fractional resistance change becomes

(11) $$\frac{\Delta R_{x}}{R_{x}}=\pi_{l}\times [\sigma_{l}^{y}-\left(\sigma_{t}^{x}+\sigma_{t}^{z}\right)]$$

The electrical sensitivity in the other directions is similarly calculated. The longitudinal piezoresistive coefficient at room temperature used for the piezoresistor is 72 × 10^−11^ Pa^−1^ as reported by Smith [29].

Due to the sensor mechanical structure and fabrication errors that affect its symmetry, plus the imperfect piezoresistors symmetrical locations on the top surface and the inherent nonlinearities of the measurement circuit, there is an error of measurement related to these factors called ‘cross-axis sensitivity’. This parameter is calculated in percentage and it is the absolute value of the fraction of the voltage output of each axis other than the one under stress and the axis under stress. For example, the cross-axis sensitivities under the *x*-axis acceleration $S_{\left(Ax-Ay\right)\%}$ and $S_{\left(Ax-Az\right)\%}$ are detected, respectively, in the output of the *Ay*, *Az*-bridge for the piezoresistors as

(12) $$S_{\left(Ax-Ay\right)\%}=S_{\left(Ay-Ax\right)\%}=\left|\frac{Vout_{Ay}}{Vout_{Ax}}\right|\%$$

(13) $$S_{\left(Ax-Az\right)\%}=\left|\frac{Vout_{Az}}{Vout_{Ax}}\right|\%$$

## 3. Results

### Performance Calculation

**Figure 5:**
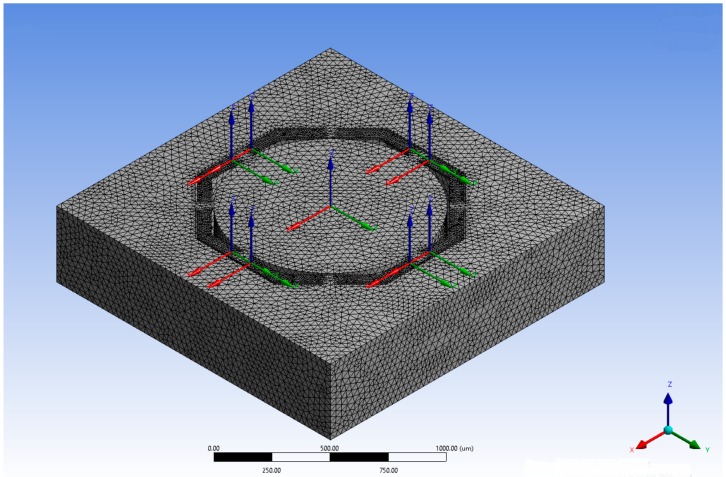
The number of nodes used for the meshing is about 250,000. As it can be seen higher number of nodes is concentrated on the beams as more accurate stress distribution is required. Moreover, the structure is fixed on the bottom frame and the load applied is an acceleration of 500 G for each axis. Notice the axial coordinates that show the piezoresistors locations.

Device performance calculations are based on an extensive stress analysis carried out by finite element method with solver available on ANSYS software package version 14.5. The material property selected from the software database is single crystal silicon anisotropic, that affects the piezoresistance effect of the piezoresistors and therefore the device sensitivity. The number of nodes used for the meshing of different designs is about 250,000, moreover, the structures are fixed on the bottom frame and the load applied is an acceleration of 500 G for each axis (Figure 5).

Equation (7) correlates the stress values to the fractional resistance change. Data of stresses are then extracted from the simulation by placing probes on the piezoresistors locations, allowing to obtain longitudinal and transversal stress values on an Excel spreadsheet, and sensitivity and cross sensitivity are eventually calculated. Sensor sensitivity is calculated using the Equation (11). Basically, from the FEA software ANSYS we obtain values of stresses (longitudinal and transversal for all three axes) exactly in the locations where the 16 piezoresistors are placed (Figure 4a and Figure 5). Using Equation (11) we obtain 16 values of resistance, one for each piezoresistor (note that the value of longitudinal piezoresistive coefficient is the one reported by Smith [29]) and with these values we calculate voltage output for each axis (see Equation (8) for Wheatstone Bridge configurations). Finally, we obtain value of sensitivity applying Equation (9), just dividing the voltage output of each axis for the applied external acceleration (500 G). Notice that the voltage in has a value of 5 V. Cross-axis sensitivity is then calculated by applying Equations (12) and (13).

In order to get the stresses values from the correct locations, a measurement circuit is developed with 16 piezoresistors located where the highest stresses are detected to maximize the sensor electrical sensitivity (see Figure 4).

**Figure 6:**
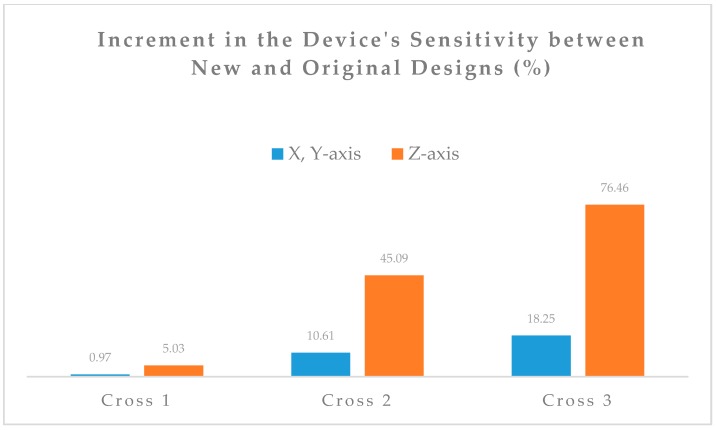
Sensitivity increment of new designs in percentage. Highest increment is for the *z*-axis sensitivity of design Cross 3 (≈80%), overall the sensitivity increases progressively from design Cross 1 to Cross 3, demonstrating the effect of MMI.

Sensitivity and cross-axis sensitivity have been calculated for the new designs under study and compared to the original circular device design. In Figure 6, the increment in the sensitivity between the new and original designs is shown.

The progressive increment of sensitivity from design Cross 1 to Cross 3 with respect to the original circular design is due to the progressive increment of the MMI, therefore the study hypothesis is confirmed.

For example, for design Cross 3, an increment in the *z*-axis of MMI of 180% determines a correspondent increment of sensitivity on the same axis of 76%.

Cross-axis sensitivity of new designs is also expected to decrease with respect to the circular design as the distribution of masses around the corners in the cross designs is reduced.

**Figure 7:**
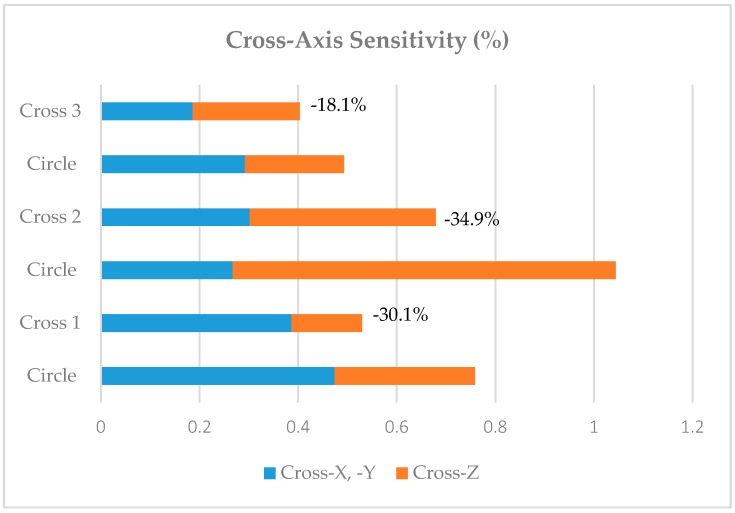
Cross-axis sensitivity reduction comparison of each new design.

Figure 7 presents the results of cross sensitivity of each new design in comparison to the original circular design. In the results, there are three different values for circle design as for each comparison the circle proof-mass volume needed to be adjusted to the same volume of the new design for simplicity of comparison. However, since the cross-axis sensitivity is given in percentage all designs can be compared accordingly.

Lowest value of cross sensitivity as expected is of the new design Cross 3, where the combined cross-X, or -Y, and -Z is of just 0.4%, well below the target of 1% for each axis.

## 4. Discussion

Comparing the optimized device performance to commercial devices, the only available three-axis medium-G accelerometer in the market, at the time of writing, are the analog 3 × 3 mm^2^ ADXL377 from Analog Devices Inc. (Norwood, MA, USA) specifically designed for concussion and head trauma detection with a range of ±200 G (used currently in IndyCar races) and the digital 3 × 3 mm^2^ H3LIS331DL from STMicroelectronics with a maximum range of ±400 G (used currently in Formula (1)). The performance comparison is presented in the Table 3. The electrical resistance of the piezoresistors used in the calculation of sensor sensitivity of this study is defined by conventional micro-scale piezoresistors at resistivity of 7.8 Ω-cm at low p-Si concentration, as reported by Smith [29]. Moreover, Smith found the p-type longitudinal piezoresistive coefficient in the [110]-direction at light boron concentrations (≈1.7 × 10^15^ cm^−3^ or resistivity of 7.8 Ω-cm) to be fairly constant at 72 × 10^−11^ Pa^−1^ [29]. This is also the longitudinal piezoresistive coefficient used in the sensitivity calculations.

The Cross 3 design developed in this study is a 1.5 × 1.5 mm^2^ device, therefore the sensitivity results reduced compared to the Analog Devices accelerometer that is 3 × 3 mm^2^. For a proper ear-plug device a 2 × 2 mm^2^ size is desirable as a bigger device would slip off the ear [18]. Moreover, the sensitivity of the ADXL377 is much higher of the device of this work as the signal output is amplified by internal circuitry, while the device developed in this work is not amplified at all.

Furthermore, the Cross 3 presents a higher measurement range because race car crash can reach impacts of more than 300 G forces. Finally, Cross 3 design presents the lower cross-axis sensitivity of all three accelerometers, therefore it is the most suitable device for biomechanical measurements. Notice that the ST device sensitivity is not comparable as the device is digital. For this device, the cross-axis sensitivity is ±2% for a range of ±70 G, therefore for impacts of ±200 G the error could reach peaks three times higher (≈±6%). Clearly this STMicroelectronics device is not suitable for biomechanical measurements as accurate measurements are necessary in case of head injuries and restraints systems design.

## 5. Conclusions

This work demonstrates the hypothesis that an increment of the MMI is a viable optimization method for a single mass mechanical structure of a piezoresistive accelerometer where high performance is a must, such as in biomechanical or biomedical applications. Examples are heart wall motion measurement for cardiac artificial pacemakers [43], hearing aid systems [44], and head injury monitoring of military soldiers in case of explosions.

The increment of sensitivity of cross design with respect to the circular design reaches 76% in the *z*-axis and 18% in the *x*- or *y*-axis. Moreover, the optimization method used allows for a simultaneous reduction of the cross-axis sensitivity down to 18.1%. These results permit a higher accuracy of measurement, which is necessary in applications that are mentioned in the first paragraph. Future work will be to manufacture the optimal design and test the performance under specified loading conditions. Moreover, a detailed mathematical modelling of correlation between sensitivity, cross-axis sensitivity, and MMI would be beneficial for future sensor design.

## Figures and Tables

**Table 1:** Piezoresistivity components for single-crystal silicon under certain doping values [29].

Piezoresistive Coefficient (10^−11^ Pa^−1^)	n-Type (Resistivity = 11.7 Ω-cm)	p-Type (Resistivity = 7.8 Ω-cm)
π_11_	−102.2	6.6
π_12_	53.4	−1.1
π_44_	−13.6	138.1

**Table 2:** Formula for transverse and longitudinal piezoresistive coefficient for various commonly encountered resistor configurations [25].

Direction of Strain	Direction of Current	Configuration	Piezoresistive Coefficient
<100>	<100>	Longitudinal	π_11_
<100>	<010>	Transversal	π_12_
<110>	<110>	Longitudinal	(π_11_ + π_12_ + π_44_)/2
<110>	<110>	Transversal	(π_11_ + π_12_ − π_44_)/2
<111>	<111>	Longitudinal	(π_11_ + 2π_12_ + π_44_)/2

**Table 3:** Performance comparison with commercial devices.

Parameter	Cross 3 (This Work)	ADXL377	H3LIS331DL
Measurement Range (G)	±500	±200	±400
Sensitivity (mV/G)	0.22	6.50	-
Cross-axis sensitivity (%FS)	<±1	±1.4	±2
Size (mm^2^)	1.5 × 1.5	3 × 3	3 × 3
